# High-Affinity Fully Human Anti-EpCAM Antibody with Biased IL-2 Exhibits Potent Antitumor Activity

**DOI:** 10.3390/biom14111399

**Published:** 2024-11-02

**Authors:** Zhi Wang, Mingkai Wang, Quanxiao Li, Yanling Wu, Tianlei Ying

**Affiliations:** 1 MOE/NHC/CAMS Key Laboratory of Medical Molecular Virology, Shanghai Frontiers Science Center of Pathogenic Microorganisms and Infection, School of Basic Medical Sciences, Shanghai Medical College, Fudan University, Shanghai 200032, China; zhiwang16@fudan.edu.cn (Z.W.); 20111010061@fudan.edu.cn (M.W.); 20111010055@fudan.edu.cn (Q.L.); 2 Shanghai Engineering Research Center for Synthetic Immunology, Shanghai 200032, China

**Keywords:** fully human antibody, EpCAM, biased IL-2 variant, immunocytokine, anti-tumor

## Abstract

Monoclonal antibodies (mAbs) are widely used in cancer therapy but often show limited efficacy for solid tumors. Enhancing anti-tumor activity by fusing cytokines to tumor-targeting mAbs, which specifically activate immune cells within the tumor microenvironment, represents a promising strategy. However, the optimal design and therapeutic efficacy of antibody–cytokine fusion formats remain unclear. The epithelial cell adhesion molecule (EpCAM), frequently overexpressed in a variety of carcinomas, serves as the target for immunotherapies. In this study, we identified a fully human mAb targeting EpCAM, designated as m801, from a previously constructed phage-displayed fully human antibody library. By fusing m801 with an IL-2 variant (IL-2v) in two configurations, m801.2 (2 anti-EpCAM Fab + 1 IL-2v) and m801.3 (1 anti-EpCAM Fab + 1 IL-2v), we identified m801.2 as the lead candidate due to its superior biophysical properties, including high thermal stability, homogeneity, and low aggregation. Furthermore, m801.2 showed strong binding affinity to EpCAM, with KD values of 0.6 nM, and an EpCAM-expressing tumor cell line, comparable to the original IgG m801. Additionally, m801.2 exhibited IL-2 receptor β subunit (IL-2Rβ)-biased binding activity, with a KD of 27.3 nM, resulting in superior effective T cell activation. In an SW480 xenograft mice model, m801.2 significantly inhibited tumor growth and demonstrated high tolerability. These findings suggest a valuable framework for the future design of immunocytokine therapies.

## 1. Introduction

The epithelial cell adhesion molecule (EpCAM, also known as CD326) is a type I transmembrane glycoprotein with an approximate molecular weight of 40 kilodaltons (kDa). Beyond its initial identification as a tumor-associated antigen, EpCAM has emerged as a key player in tumor development, acting as a signaling receptor and activator of the Wnt pathway, which is involved in cell proliferation, migration, and invasiveness [1,2,3]. Studies have shown that EpCAM is overexpressed in a wide range of human carcinomas, including pancreas, colorectal, and prostate cancers, as well as in tumor-initiating cells. Due to the high expression and antigenicity, EpCAM has been considered as a prime target for monoclonal antibody (mAb) therapies [4].

Although numerous anti-EpCAM mAbs have been developed and tested in clinical studies, many early candidates, such as adecatumumab [5] and edrecolomab [6], showed limited clinical benefit and raised safety concerns for cancer patients [7,8]. These limitations have promoted the exploration of more advanced therapeutic strategies. More recently, a bispecific EpCAM/CD3-antibody, antibody–drug conjugates (ADCs), and an antibody–cytokine fusion protein (termed immunocytokine) have emerged as promising EpCAM-targeting approaches for cancer treatment [9]. These antibody-based therapeutics deliver drugs or cytokines directly to the tumor via EpCAM-targeting antibodies, enabling ADCs or immunocytokines to specifically kill tumor cells or activate immune cells within the tumor microenvironment, thereby offering enhanced efficacy and safety. Tucotuzumab celmoleukin (huKS-IL2), an immunocytokine that acts by genetically fusing interleukin-2 (IL-2) to a humanized monoclonal antibody against EpCAM [10], has demonstrated safety and immunologic activity in cancer patients [11]. Additionally, the combination of tucotuzumab celmoleukin with radiofrequency ablation enhanced anti-tumor effects and immunologic memory in murine colon cancer [12].

Interleukin-2 (IL-2), initially identified as a T cell growth factor [13,14], plays a dual role in the immune system by stimulating the activation of cytotoxic effector cells and driving the expansion of regulatory T cells (Treg) [15,16,17,18,19]. IL-2 exerts its biological functions by interacting with its IL-2 receptor (IL-2R), which is composed of three subunits: IL-2Rα (CD25), IL-2Rβ (CD122), and IL-2Rγ (CD132) [20,21]. Effector cells, like CD8+ T and natural killer (NK) cells, predominantly express high levels of dimeric IL-2Rβγ, while suppressive Treg cells and endothelial cells express high IL-2Rα [22]. IL-2 binding to IL-2Rα has been associated with vascular leak syndrome and the preferential expansion of Treg cells, both of which can limit its therapeutic efficacy. Therefore, IL-2 has been engineered to selectively bind to IL-2Rβγ without recognizing IL-2Rα. Roche has developed a new class of IL-2 variants (IL-2v) with biased binding properties, some of which are additionally targeted to cell-surface proteins overexpressed in tumors or the surrounding stroma to enhance local tumor retention, such as CEA-IL2v [23] and FAP-IL2v [24], which have shown improved therapeutic efficacy. However, the anti-tumor effects of combining anti-EpCAM antibodies with IL-2v remain unclear.

Previously, we generated a fully human antibody phage-displayed Fab library with a diversity of 1.5 × 10^11^ derived from 40 healthy volunteers, from which a variety of high-affinity mAbs were identified [25,26,27,28]. In this work, we utilized this platform to discover a fully human mAb targeting EpCAM that binds with high affinity to both human and mouse EpCAM. To determine the optimal form of IL-2v and antibody fusion with favorable properties and biological activity, we constructed two forms: m801.2 (IL-2v fused at the C-terminus of IgG1 Fc, 2 anti-EpCAM Fab + 1 IL-2v) and m801.3 (IL-2v fused at the N-terminus, 1 anti-EpCAM Fab + 1 IL-2v). Compared to m801.3, m801.2 exhibits superior biophysical properties and greater potency in inhibiting tumor growth. These findings offer a promising approach to overcoming the limitations of traditional mAbs and cytokine therapies in cancer treatment.

## 2. Materials and Methods

### 2.1. Cell Lines

The Expi293 cell line (#A14527, Thermo Fisher, Waltham, MA, USA) was purchased and maintained in an Expi293 expression medium. The human colon cancer cell lines SW480 and HCT116 were generously provided by the Institute of Radiology, Fudan University. Both cell lines were cultured in Dulbecco’s Modified Eagle Medium (DMEM) supplemented with 10% fetal bovine serum (FBS, Gibco BRL, Grand Island, NY, USA) and 1% penicillin-streptomycin (#03-031-1B, Biological Industries, Beijing, China) in a humidified incubator at 37 °C with 5% CO_2_. All cell lines were regularly tested for mycoplasma contamination and authenticated using short tandem repeat (STR) profiling to ensure experimental validity. Human PBMCs were purchased from the OriBiotech Co., Ltd. (Shanghai, China).

### 2.2. Identification of Fully Human Monoclonal Antibody Against EpCAM

To identify anti-EpCAM antibodies, the biotinylated human EpCAM protein (ACROBiosystems, Newark, DE, USA) was used as an antigen for the panning of the phage-displayed naïve human antibody library, as described previously [29,30]. Four rounds of screening were conducted, and the enrichment of antigen-specific phages was assessed using a polyclonal enzyme-linked immunosorbent assay (ELISA). A total of about 400 single clones from the enriched rounds were randomly picked and determined for binding to EpCAM by ELISA. Positive clones selected out of these were produced as purified Fab or converted into the full-length human IgG1 format.

For Fab expression, Escherichia coli (*E. coli*) HB2151 were used, and purification was performed via Ni-NTA affinity chromatography. Briefly, the recombinant plasmids carrying a C-terminal hexahistidine and FLAG tag were transfected into HB2151 bacteria. After induction with 1 mM isopropyl β-D-1-thiogalactopyranoside (IPTG) (Yeasen, Shanghai, China) at 30 °C for 14 h, the E. coli cells were harvested and resuspended in phosphate-buffered saline solution (PBS) containing 500 mM NaCl. Cells were lysed by ultrasonication (JL-150W, Jinlan Instrument Manufacturing Co., Ltd., Shanghai, China) at 0 °C, and the lysate was centrifuged at 7500× *g* for 20 min to collect the supernatant. Fab fragments were purified by Ni-NTA resin (#20503ES10, Yeasen, Shanghai, China) according to the manufacturer’s instructions. The purified fractions were immediately buffer exchanged into PBS and concentrated using an Amicon ultra centrifugal concentrator (Merck Millipore, Billerica, MA, USA) with a molecular weight cut-off of 10 kDa. Protein purity was evaluated by sodium dodecyl sulfate polyacrylamide gel electrophoresis (SDS-PAGE), and the protein concentration was determined using the NanoDrop 2000 spectrophotometer (Thermo Fisher, Waltham, MA, USA).

### 2.3. Expression and Purification of IgG Antibody

To convert and prepare IgG antibody, the heavy and light chains of the Fabs were amplified and cloned into the PTT-IgG1 vector, formatted for human IgG1. The recombinant plasmids were verified by sequencing and then transfected into Expi293F for transient expression using Polyethylenimine (PEI). After 5 days, the supernatants were harvested and incubated overnight with Protein A resin (GenScript, Piscatawa, NJ, USA). The resin was collected on a chromatography column, washed with PBS (pH 7.4), and the antibody was eluted in 0.1 M glycine (pH 2.7). The eluates were neutralized using 1 M Tris-HCl (pH 9.0), and the buffer was immediately replaced with PBS (pH 7.4) for further analysis.

### 2.4. Knob-Into-Hole-Based Immunocytokines

The immunocytokines were constructed using a knob-into-hole [31] strategy to facilitate heterodimerization. Site-directed mutagenesis was performed on the Fc region of the PTT-IgG1 vector to generate either a knob (T366W) or a hole (T366S, L368A, and Y407V). The gene encoding the IL-2 variant (IL-2v) was synthesized by GenScript and subsequently cloned into either the N-terminus or C-terminus of the knob Fc region, resulting in different antibody–cytokine formats.

The recombinant constructs were verified by sequencing and transfected into Expi293F cells using PEI for transient expression. After 5 days of culturing, the supernatants were harvested and the immunocytokines were purified using Protein A resin as described previously. This knob-into-hole design allowed for the efficient production of stable immunocytokines for further analysis.

### 2.5. ELISA

A 96-well half-area microplate (#3690, Corning, Corning, NY, USA) was coated with 100 ng of EpCAM antigen per well and incubated overnight at 4 °C. After washing three times with 300 μL of PBST (PBS with 0.05% Tween 20), the plate was blocked with PBS containing 5% skim milk (*w*/*v*) (#36120ES76, Yeasen, Shanghai, China) for 1 h at 37 °C. Then, the plate was washed by PBST three times and incubated with 50 μL of three-fold serially diluted antibody solutions per well for 1.5 h at 37 °C. After PBST washing three times, the 1:10,000 diluted anti-FLAG M2-HRP monoclonal antibody (#A8592-1MG, Sigma-Aldrich, St. Louis, MO, USA) was added to detect the bound antibodies, and binding activity was determined by measuring the absorbance at 405 nm after the addition of 50 μL ABTS substrate and 3–5 min incubation at room temperature (#002024, Thermo Fisher, Waltham, MA, USA).

### 2.6. Size-Exclusion Chromatography–High-Performance Liquid Chromatography (SEC-HPLC)

The purified antibodies were analyzed by SEC-HPLC. Each antibody was diluted to a concentration of 0.5 mg/mL in sterile 1× PBS buffer, with at a volume of 80–100 μL, and then injected into a ChromCoreTM C18 column on a SUPER High Performance Liquid Chromatography device (Waters 2695, Milford, MA, USA). All samples were passed through the column at a flow rate of 0.5 mL/min using 1× PBS as the mobile phase buffer. Analysis times ranged from 30 to 60 min per sample.

### 2.7. Dynamic Light Scattering (DLS)

The antibodies were prepared by centrifuging at 11,300× *g* for 10 min, filtered through a 0.22 μm filter (Merck Millipore, Billerica, MA, USA), and adjusted to a concentration of 1 mg/mL. The size distribution of protein particles was measured in polystyrene cuvettes at 25 °C using a Zetasizer Nano ZSZEN 3600 (Malvern Instruments Ltd., Worcestershire, UK). The data of three independent experiments were collected.

### 2.8. Thermal Stability of Immunocytokines Using Circular Dichroism (CD) Spectroscopic Analysis

The CD spectra of antibodies were collected using a J-815 spectropolarimeter (Jasco International, Tokyo, Japan). Antibodies were prepared at a concentration of 0.25 mg/mL in PBS. The molar ellipticity signals were recorded at 220 nm. The samples were heated from 20 °C to 95 °C at a rate of 1 °C/min. Data analysis was performed by GraphPad Prism, and the thermal denaturation (Tm) values were extrapolated by fitting the data to a Boltzmann sigmoidal curve. The area under the curve (AUC) was calculated.

### 2.9. Bio-Layer Interferometry (BLI) Binding Assay

The binding kinetics of antibodies or antibody–cytokine protein to EpCAM or IL-2R were measured by BLI on an Octet-RED96 (ForteBio, Fremont, CA, USA). A biotinylated EpCAM or IL-2R antigen was loaded onto streptavidin-coated (SA) biosensors (#18-5019, Sartorius, Hamburg, Germany) at 10 μg/mL, followed by incubation with three-fold serially diluted antibodies starting at 100 nM in PBST (PBS with 0.02% Tween 20) for 400–1200 s for association, and then immersed into PBST for another 400–1200 s for dissociation. All the curves were fitted by a 1:1 binding model using the Data Analysis software (Version 10.0, ForteBio, Fremont, CA, USA). *K*_D_ values were determined with R^2^ values of greater than 95% confidence level.

### 2.10. Flow Cytometry

To determine the EpCAM-binding capacity of the antibodies, 1 × 10^6^ HCT116 cancer cells were incubated with indicated concentrations of antibodies for 1 h on ice, followed by washing twice with 100 μL of 2% FPBS (PBS with 2% Fetal cattle serum). For antibody–cytokines, SW480 cells (1 × 10^5^ per well) were seeded in 96-well cell plates and incubated with three-fold serially diluted antibodies. The cells were incubated with FITC-conjugated anti-human IgG Fc antibody (#62-8411, Thermo Fisher Scientific, Waltham, MA, USA) and analyzed by BD FACSCalibur (BD Biosciences, San Jose, CA, USA) or Attune NxT flow cytometer (Thermo Fisher Scientific, Waltham, MA, USA). The data analyses were performed with Flow Jo (Version 10.4, BD biosciences, San Jose, CA, USA).

### 2.11. Immune Cell Activation Assay

Human PBMCs were cultured in an RPMI-1640 medium and seeded into 96-well cell plates at a density of 1 × 10^6^ cells/well. The antibodies were serially diluted by the medium at the initial concentration of 100 nmol/L. A total of 100 μL of antibody solution was added to each well and incubated for 6 h. The cells were washed with 2% FPBS 3 times and resuspended with 100 μL 2% FPBS containing 0.1 μL anti-CD69, conjugated with AlexaFluor 647 (Thermo Fisher Scientific, Waltham, MA, USA). After being incubated on ice for 45 min away from light, the cells were gently washed, fixed with 100 μL 4% paraformaldehyde, and analyzed by flow cytometry (FCM). Data were processed by Flow Jo (Version 10.4, BD biosciences, San Jose, CA, USA).

The level of IFN-γ secretion directly reflects the activation level of immune cells. In brief, PBMC were incubated with increasing concentration of antibodies for 72 h. Supernatant from the assay plates was collected to determine the IFN-γ concentration by IFN-γ ELISA test kit (#DIF50C, R&D Systems, Minneapolis, MN, USA) according to the manufacturer’s instructions. All samples were tested in triplicates. The data were nonlinearly fitted, and the half-maximal effective concentrations (EC_50_) were calculated via regression of the four parameters using GraphPad Prism (Version 8.2.1, GraphPad Software, San Diego, CA, USA).

### 2.12. Animal Study

All of the procedures related to animal handling, care, and treatment were performed and approved by the ethics committee of the School of Basic Medical Sciences at Fudan University.

Female 6-week-old NSG (severe immunodeficiency) mice were purchased from the Southern Model Biotechnology Co., Ltd. (Shanghai, China) and subcutaneously inoculated with 5 × 10^6^ of SW480 cells and PBMC mixture on the right flank. To reconstitute PBMC immune system in NSG mice, 5 × 10^6^ human PBMC was injected intravenously into mice tail every 7 days. When the tumor volume reached appropriately 200 mm^3^, the mice were randomized into seven groups (n = 4–5 mice per group) and received intraperitoneal (i.p.) injections of various antibodies, including IgG1 CR3022 (mAb against SARS-CoV-2, used as isotype control), IL-2v, m801, m801 combined with IL-2v, m801.2, and m801.3. The received antibodies were at a dosage of 4 mg/kg. The tumor volumes and body weights of mice were monitored every 3 days. Tumor volumes were measured with an electronic caliper and calculated using the formula: 1/2 × (length × width^2^). At the end of the experiment, all mice were scarified and tumors were harvested for weighting and photos.

### 2.13. Statistical Analyses

Statistical analyses were conducted using Prism software (Version 8.2.1, GraphPad Software, San Diego, CA, USA). Data were presented as mean ± SD from three independent experiments. Individual or multiple group comparisons were performed by the two-tailed unpaired Student’s *t* test. A statistically significant difference was defined as *p* < 0.05 and different levels of significance were set as * *p* < 0.05, ** *p* < 0.01, *** *p* < 0.001.

## 3. Results

### 3.1. Identification of Anti-EpCAM Antibody m801 (E3) with High Affinity

**Figure 1:**
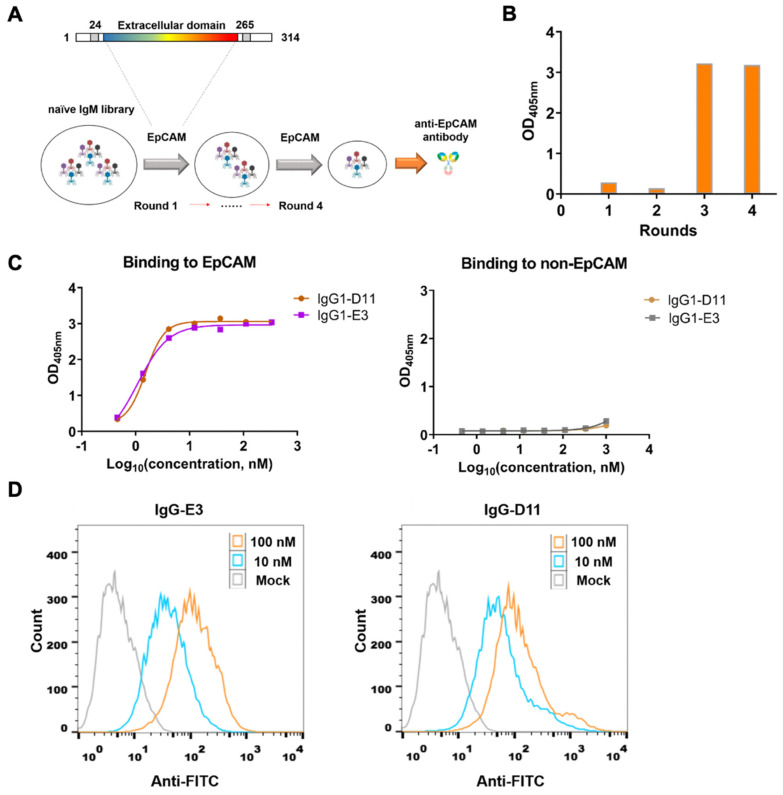
Identification and characterization of high-affinity anti-EpCAM antibodies. (**A**) Schematic representation of the biopanning process using a phage-displayed Fab library against the EpCAM extracellular domain (ECD). Four rounds of panning were conducted to enrich EpCAM-specific phages. (**B**) Polyclonal ELISA results showing significant enrichment of EpCAM-specific binding phages in rounds 3 and 4. (**C**) ELISA binding curves of IgG1-D11 and IgG1-E3 to EpCAM (**left**) and a control protein (**right**). The control protein is the envelope protein domain III of Zika virus. (**D**) Cell binding of anti-EpCAM mAbs (10 nM and 100 nM) to human colon cancer cell line HCT116, as determined by flow cytometry.

To obtain the specific antibody against EpCAM, we utilized a large phage-displayed naive Fab antibody library for biopanning. The extracellular domain (ECD) of EpCAM was used as the target antigen, and four rounds of bio-panning were conducted (Figure 1A). Polyclonal phage ELISA results demonstrated a significant enrichment of EpCAM specific-binding phages in round 3 and round 4 (Figure 1B). A total of 400 clones were selected from these rounds, and positive clones with at least five-fold higher binding activity than controls were identified by soluble monoclonal ELISA. Sequencing of these clones revealed multiple families based on their amino acid sequence diversity in the complementarity-determining region (CDR) 3 of the VH or VL genes. Two unique antibody candidates, D11 and E3, were selected for further expression. Both antibodies exhibited strong binding ability to EpCAM in Fab and IgG1 formats (Supplementary Figure S1, Figure 1C), with EC_50_ values of 1.53 nM for IgG1-D11 and 1.09 nM for IgG1-E3, respectively (Figure 1C). No binding activity was observed against non-EpCAM proteins, confirming specificity. Moreover, antibody E3 showed stronger binding to the human colon cancer cell line HCT116, which is a well-known EpCAM-expressing line [32] (Figure 1D). Based on these results, antibody E3 was selected for further analysis and designated as m801 in subsequent studies.

Immunogenetic analysis of their sequences was performed using the IMGT tool to determine the closest VH and VL germline genes (Table 1). Analyses of germline gene usage indicated that they originated from different B-cell lineages. Furthermore, their nucleotide sequences displayed more than 95% IGHV identity to the germline (Table 1). Therefore, both antibodies were germline-like mAbs, which in general exhibit lower immunogenicity and better druggability properties compared to somatically hypermutated antibodies.

### 3.2. Engineering and Characterization of Anti-EpCAM-IL-2v Immunocytokines

**Figure 2:**
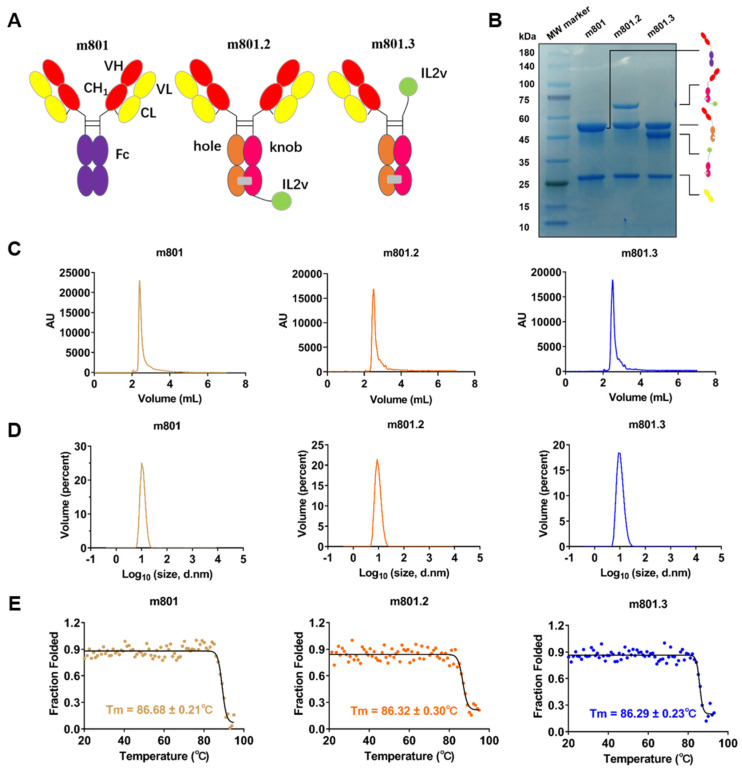
Design, expression, and biophysical characterization of anti-EpCAM-IL-2v immunocytokines. (**A**) Schematic illustration of the structural design of m801, m801.2, and m801.3 fusion proteins. The Fc region of mAb m801 introduced knob-into-hole (KIH) mutations; the IL-2v was fused to the C-terminus of the Fc region in m801.2, and to the N-terminus of the Fc region in m801.3. (**B**) SDS-PAGE analysis of m801, m801.2, and m801.3 under reducing conditions. The bands correspond to the expected molecular weights of the antibody constructs. SDS-PAGE original images can be found in Supplementary Materials. (**C**) Size-exclusion chromatography (SEC-HPLC) profiles of m801, m801.2, and m801.3. (**D**) Dynamic light scattering (DLS) analysis of m801, m801.2 and m801.3. (**E**) The melting temperature (T_m_) values of m801, m801.2, and m801.3, as measured by CD spectroscopy.

In previous studies, monotherapy with anti-EpCAM antibodies demonstrated limited anti-tumor efficacy, highlighting the need for a novel approach to enhance therapeutic outcomes [33]. One promising strategy is fusing antibodies with cytokines, such as IL-2, to potentiate immune responses against tumors. The majority of IL-2-based therapeutics currently being evaluated in clinical trials utilize an IL-2Rβ/γ-biased IL-2 strategy, with the intent of reducing systemic IL-2 toxicity and selectively activating IL-2Rβ/γ-expressing CD8+ T cells and NK cells over IL-2Ra/b/g-expressing endothelial cells and Tregs [34]. Therefore, we designed the antibody–cytokine fusion protein composed of anti-EpCAM mAb and an IL-2Rβ/γ-biased IL-2 variant (IL-2v) and explored the structural format on their performances. Specifically, the IL-2v moiety was fused to either the N-terminus or C-terminus of anti-EpCAM mAb m801, resulting in two constructs, m801.2 and m801.3, respectively (Figure 2A). Both of these antibodies were successfully expressed using a mammalian cell system with high yields exceeding 10 mg/L, and showed high purity in SDS-PAGE (Figure 2B). To analyze the aggregation and degradation of antibodies, we performed analytic size-exclusion–high-performance liquid chromatography (SEC-HPLC). One predominant peak, with an area exceeding 95%, was observed for m801, m801.2 and m801.3, indicating high purity and stability (Figure 2C). Dynamic light scattering (DLS) further confirmed the monomeric nature and homogeneity of antibodies, with no significant aggregation observed (Figure 2D). Furthermore, thermodynamic stability was assessed using circular dichroism (CD) spectroscopy. As shown in Figure 2E, the melting temperature (T_m_) values of m801.2 (86.32 ± 0.30 °C) and m801.3 (86.29 ± 0.23 °C) were similar to the value of m801 (86.68 ± 0.21 °C), demonstrating that the fusion of IL-2v did not compromise the structural stability of the antibodies (Figure 2E). The overall protein stability was further evaluated by comparing the area under the curve (AUC) of the stability profiles. Analysis revealed that m801.2 exhibited a higher AUC than m801.3 (Supplementary Figure S2), consistent with the Tm values.

### 3.3. The Immunocytokines Exhibit Strong Binding Activity to EpCAM

**Figure 3:**
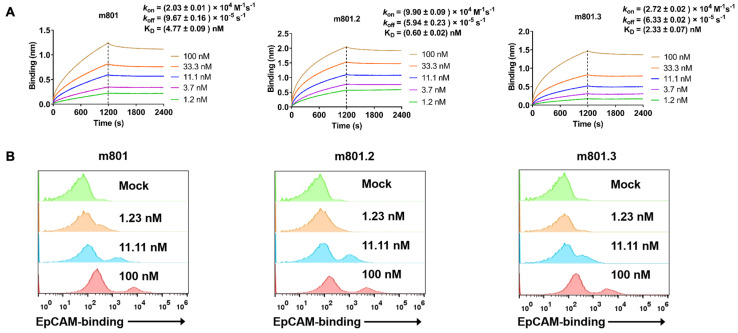
Binding kinetics of anti-EpCAM-IL-2v to EpCAM and the EpCAM-expressing cell line. (**A**) The binding affinities of m801, m801.2, and m801.3 to human EpCAM, as measured by BLI. The vertical dashed line corresponds to the transition between the association and dissociation phases. Curve fitting was performed to extrapolate equilibrium dissociation constant values using a 1:1 global model. (**B**) Flow cytometry of m801, m801.2, and m801.3 against SW480 cells. The SW480 cells were incubated with m801, m801.2, and m801.3 at the indicated concentration.

To assess the functional integrity of the anti-EpCAM-IL-2v fusion constructs, bio-layer interferometry (BLI) assays were performed to evaluate the binding kinetics of the immunocytokines to EpCAM. The results demonstrated that m801.3 (K_D_ = 2.33 nM) exhibited a similar EpCAM-binding affinity to the parental m801 (K_D_ = 4.77 nM). Notably, m801.2 showed the stronger affinity for human EpCAM, with a K_D_ of 0.60 nM, attributed to its rapid association rate (*kon* = 9.90 × 10^4^ M^−1^s^−1^) and remarkably slow dissociation rate (*koff* = 5.94 × 10^−5^ s^−1^) (Figure 3A). Comparable binding profiles were observed with mouse EpCAM, where m801.2 also exhibited the highest binding affinity, with a K_D_ of 3.76 nM (Supplementary Figure S3).

Next, we employed flow cytometry analysis using SW480 cells, which are known as EpCAM expressing cell lines. The results indicated that m801 and m801.2 have a similar affinity, m801.3 has moderate affinity due to its monovalent EpCAM-binding arm (Figure 3B).

### 3.4. Immune Cell Binding and Activation by Anti-EpCAM-IL-2v Immunocytokines

**Figure 4:**
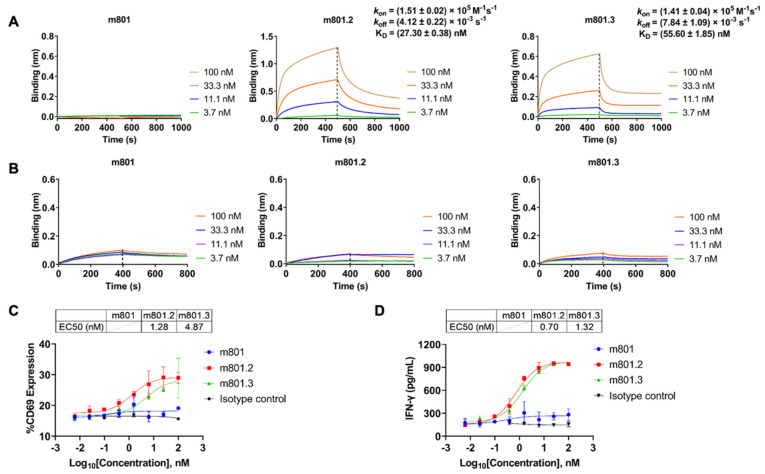
Anti-EpCAM-IL-2v immunocytokines show potent activation of immune cells in vitro. (**A**,**B**) Binding kinetics of m801, m801.2, and m801.3 to the immobilized IL-2Rβ (**A**) or IL-2Rα (**B**) as determined by biolayer interferometry. The antibody concentrations used were 100 nM (orange), 33.3 nM (blue), 11.1 nM (purple), and 3.7 nM (green). (**C**) Flow cytometry analysis of CD69 expression in effector cells treated with varying concentrations of m801, m801.2, and m801.3. Isotype control: mAb CR3022. (**D**) IFN-γ secretion levels in PBMC culture supernatants following stimulation with varying concentrations of anti-EpCAM-IL-2v immunocytokines or control antibodies. Each data point is shown as mean ± SD of three biological replicates.

We next investigated the IL-2R binding profiles of anti-EpCAM-IL-2v immunocytokines. Both m801.2 and m801.3 exhibited strong binding to the IL-2Rβ subunit (Figure 4A). As expected, neither antibody displayed binding activity toward the IL-2Rα subunit (Figure 4B), confirming the IL-2Rβ biased-targeting of designed IL-2v. The parental antibody m801 showed no binding activity to either IL-2Rβ or IL-2Rα, as it lacks IL-2v.

To further explore the immune cell activation potential of anti-EpCAM-IL-2v immunocytokines, we utilized human PBMCs and measured the expression level of CD69. CD69, a classical early marker of lymphocyte cell activation, is typically upregulated within 2–3 h following T cell or NK cell activation [35]. We observed that both m801.2 and m801.3 efficiently upregulated CD69 expression in a dose-dependent manner, with EC_50_ values of 1.28 nM for m801.2 and 4.87 nM for m801.3, indicating the superior activity of m801.2 (Figure 4C). In contrast, mAb m801 and isotype control antibody groups showed negligible CD69 upregulation across all tested concentrations (0.01–100 nM).

Furthermore, we assessed T cell activation by measuring IFN-γ secretion from PBMC stimulated with varying concentrations of anti-EpCAM-IL-2v immunocytokines or control mAb. As shown in Figure 4D, both m801.2 and m801.3 induced significant, dose-dependent increases in IFN-γ production. The EC_50_ values again demonstrated the superior potency of m801.2, which may be contributed to the long and flexible linker between the IL-2v domain and the IgG constant region of m801.3, and may affect the stability of IL-2v binding to the receptor (Supplementary Figure S4). These results indicated that the fusion of IL-2v to the m801 antibody enhances the activation of immune cells, particularly T cells, leading to potent immune responses.

### 3.5. In Vivo Anti-Tumor Efficacy of Anti-EpCAM-IL-2v Immunocytokines

**Figure 5:**
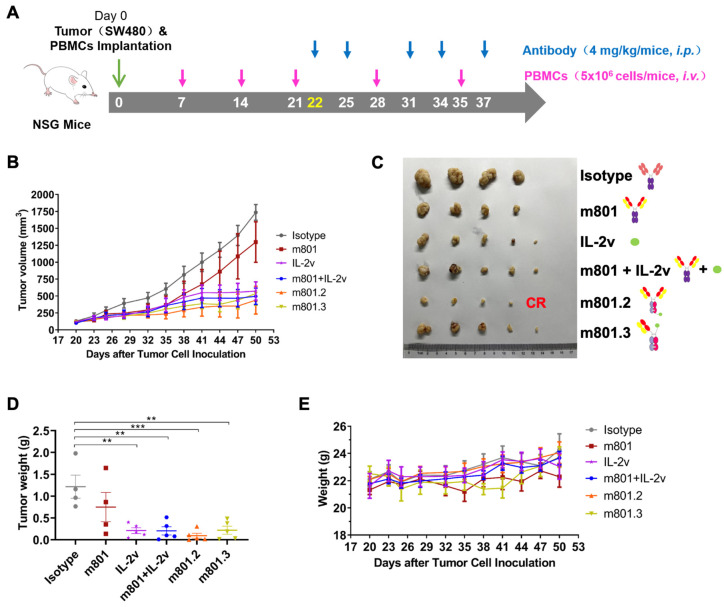
In vivo anti-tumor efficacy of the anti-EpCAM/IL-2v in mice. (**A**) NSG mice bearing subcutaneously established SW480 xenograft tumors were treated intraperitoneally with indicated proteins (blue) and intravenously 5 × 10^6^ PBMCs (pink) on the specified days. (**B**) Tumor growth curves. Tumor volumes were monitored every 3 days and calculated as (length × width^2^)/2. Data are shown as mean ± SEM. (**C**) Photographs of excised tumor. The tumor of one mouse in m801.2 group disappeared, marked with “CR”. (**D**) Tumor weight of mice. Data are shown as mean ± SEM. Statistical significance was determined by one-way ANOVA with Tukey’s multiple comparisons tests (**, *p* < 0.01; ***, *p* < 0.001). (**E**) Body weight change in mice. Data are shown as mean ± SEM.

To further evaluate whether the immunocytokines exhibit enhanced anti-tumor efficacy, we compared their anti-tumor efficacy using the SW480 colon carcinoma model in human PBMC-reconstituted mice. The colon cancer cells SW480 and PBMC mixture were subcutaneously inoculated into severely immunodeficient NSG mice. Once the tumors reached a measurable size (~200 mm^3^), mice received intraperitoneal treatments with m801 monotherapy, IL-2v monotherapy, m801.2, m801.3 and a combination of m801 and IL-2v, administered twice weekly at a dose of 4 mg/kg (Figure 5A). PBMCs were intravenously injected weekly throughout the experiment.

The tumor growth curves for six groups have been shown in Figure 5B. Compared to the control group, all treatment groups, except for the m801 monotherapy group, significantly inhibited tumor growth (Figure 5B). Among these, m801.2 exhibited the most evident efficacy, achieving a tumor growth inhibition rate of 74.72%. Notable, by day 50, the tumor volumes in the m801.2 group were smaller than those in other groups, with one mouse showing complete tumor regression (Figure 5C,D). The toxicity of drugs was evaluated by monitoring changes in body weight. Obviously, the body weight of mice in all treatment groups remained stable, with no significant differences compared to isotype control group, indicating the high tolerability of the treatments with no obvious signs of toxicity (Figure 5E).

## 4. Discussion

Despite being the key and effective therapeutics for human cancer, monoclonal antibodies (mAbs) against the tumor-associated antigen EpCAM have shown a limited clinical benefit that prevented their broader use in the clinic. Edrecolomab (Panorex^®^) is the first monoclonal EpCAM-specific therapeutic antibody [36]. However, the clinical effects of this mouse IgG2a mAb is limited in various adenocarcinomas [37,38], likely due to its low affinity for tumors (K_D_ = 1530 nM) and high immunogenicity associated with its mice origin [39]. The human-engineered and humanized antibodies, ING-1 and 3622W94, demonstrated higher affinity (approximately 0.16–0.19 nM) but were associated with severe toxicities, including acute pancreatitis [40,41]. A novel human IgG1 anti-EpCAM antibody, adecatumumab (MT201), exhibited dose-dependent anti-tumor activities and hightolerance for metastatic breast cancer (MBC), primarily through effector functions such as antibody-dependent cellular cytotoxicity (ADCC) and complement-dependent cytotoxicity (CDC) [7]. This highlights the potential of generating fully human IgG1 antibodies, which retain high affinity and low immunogenicity, as a more promising therapeutic approach. Notably, germline-like antibodies, generated using the large naïve antibody library-based technologies, have revolutionized the rapid development of human antibodies with specific binding activity and desirable properties [29,31]. Herein, through the bio-panning of a human Fab library with human EpCAM, we successfully selected and characterized a panel of novel germline-like human mAbs with binding affinity and specificity against human EpCAM. Since germline mAbs typically exhibit good druggability and low to no immunogenicity, in particular the mAb with an affinity of 4.77 nM, m801, they have potential for further development as antibody-based therapeutics.

It is worth noting that underivatized mAb may not be sufficient for effective cancer therapy; as reflected in our findings, alternative approaches such as bispecific anti-EpCAM/anti-CD3 antibodies and ADCs face challenges with stability, specificity, and toxicity in clinical trials. Immunocytokines, which combine antibodies with superior tumor-targeting ability and cytokines with broad-based immune-modulatory activities, effectively mobilize or sustain anti-tumor immune responses at the tumor site. In our study, we found that immunocytokines, regardless of whether they are C-terminus or N-terminus-linked cytokines, exhibited similar biophysical properties to mAb, including high yields, purity, thermal stability, low aggregation rate, and uniformity. Both the SEC-HPLC and DLS analyses showed a single peak and the melting temperature values were above 85 °C for both fusion formats. However, the biological functions differed significantly depending on the cytokine fusion site. When the cytokine was fused to the C-terminus, the antibody maintained its bivalency and avidity, leading to a stronger tumor-binding affinity. In contrast, when the cytokine was fused to the N-terminus, the monovalent configuration of the antibody weakened its affinity. Since cytokine–receptor binding is generally weaker than antigen–antibody binding, positioning the antibody to accumulate at the tumor site and subsequently activate immune cells in the tumor microenvironment is crucial. Our in vitro and in vivo studies demonstrated that C-terminal cytokine fusion resulted in higher immune activity and stronger anti-tumor efficacy.

IL-2-based immunotherapies have shown great potential in cancer treatment, with several strategies currently in development to achieve immunostimulation without activating suppressive Tregs, including IL-2 muteins, PEGylated IL-2, IL-2/anti-IL-2 Ab complex, and IL-2-CD25 fusion proteins. For example, NKTR-214, a wild-type IL-2 prodrug conjugated to releasable polyethylene glycol (PEG) chains, preferentially binds to IL-2Rβγ over CD25, thereby shifting the ratio of T cells toward CD8+ T cells. Roche also developed an IL-2 variant (IL-2v) harboring F42A, Y45A, and L72G mutations to improve CD122 selectivity. IL-2v has been fused with several mAbs targeting PD-1 [42], carcinoembryonic antigen (CEA) [23], and fibroblast activation protein alpha (FAP), effectively targeting PD-1+ T cells, tumor cells, and cancer-associated fibroblasts, respectively. These IL-2 muteins were more effective than wild-type IL-2 (IL-2wt) at stimulating CD8 +  T cells and NK cells and inducing antitumor activity in preclinical tumor studies. Despite the significant antitumor effects observed in preclinical studies, IL-2 variants targeting intermediate-affinity IL-2R have yielded disappointing results in clinical trials, as exemplified by the PIVOT IO-001 phase 3 trial of NKTR-214 by Nektar and Bristol Myers Squibb [43]. These results underscore the need for more refined drug design strategies to improve clinical outcomes. Recent studies have revealed that strategies targeting trimeric high-affinity IL-2 receptors, particularly in combination with PD-1 blockade, have shown promising therapeutic efficacy [44].

Taken together, we developed a high-affinity, fully human monoclonal antibody targeting EpCAM and fused it with IL-2v to construct immunocytokines (termed as m801.2 and m801.3). Compared to m801.3, m801.2 demonstrated favorable biophysical properties, including high stability and low aggregation, as well as a strong binding affinity to EpCAM and IL-2Rβ, leading to more effective T cell activation. In an SW480 xenograft model, m801.2 significantly inhibited tumor growth with high tolerability. These findings highlight a full-length mAb and one cytokine as a promising approach for advancing immunocytokine therapies in cancer treatment.

## 5. Conclusions

In conclusion, our research revealed that a novel germline-like fully human antibody with nanomolar binding affinity against EpCAM, fused with a biased IL-2v, exhibits potent anti-tumor activities.

## 6. Patents

Y.W. and T.Y. are listed as inventors on one patent application related to this work.

## Figures and Tables

**Table 1:** Genetic analysis of the heavy and light chain variable regions of EpCAM-specific antibodies.

mAb	Variable Region	Identity (%)	D	J	CDR3
*V_H_*					
E3	HV3-30-5*03	98.26	D6-25*01	J3*02	ARGLPARAFDI
D11	HV3-23*04	95.83	D1-26*01	J4*02	ASASGTYHANY
*V_L_*					
E3	KV1-12*01	96.77		J4*01	QQANSFPLT
D11	LV3-25*02	99.28		J2*01	QSADSSGTYVV

## Data Availability

Data are available upon request from the corresponding authors.

## Supplementary Materials

The following supporting information can be downloaded at: https://www.mdpi.com/article/10.3390/biom14111399/s1. Figure S1: Expression and binding activity of anti-EpCAM Fabs from phage-displayed Fab antibody library; Figure S2: The area under the stability curve; Figure S3: The binding affinities of m801, m801.2, and m801.3 to mouse EpCAM, as measured by BLI; Figure S4: Molecular dynamics simulations for m801.2 and m801.3 with IL-2Rβ/γ complex.
