# Supplementary material for: High-Affinity Fully Human Anti-EpCAM Antibody with Biased IL-2 Exhibits Potent Antitumor Activity
Source: Biomolecules. 2024 Nov 2;14(11):1399. doi: 10.3390/biom14111399 (PMC11591715; doi:10.3390/biom14111399)

Supplementary Materials for

High-affinity fully-human anti-EpCAM antibody with biased-IL-2 exhibits potent antitumor activity

Zhi Wang [1#], Mingkai Wang [1#], Quanxiao Li [1], Yanling Wu [1,2,*] and Tianlei Ying [1,2,*]

*Corresponding author: yanlingwu@fudan.edu.cn (Y.W.); tlying@fudan.edu.cn (T.Y.)

This PDF file includes:

Figures S1 to S4
Original images

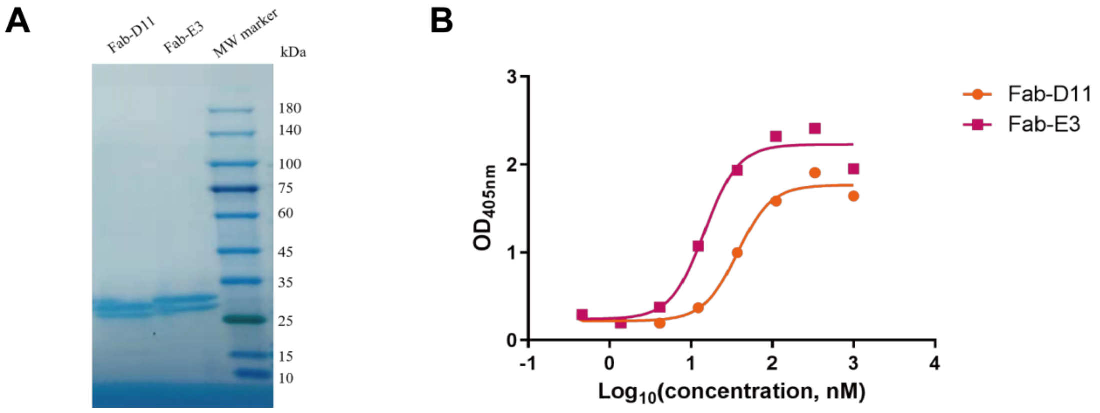

Figure S1 Expression and binding activity of anti-EpCAM Fabs from phage-displayed Fab antibody library.

A. SDS-PAGE analysis of the integrity and purity of the Fab.

B. Binding capacity of Fab-D11 and Fab-E3 to human EpCAM antigen, as measured by ELISA.

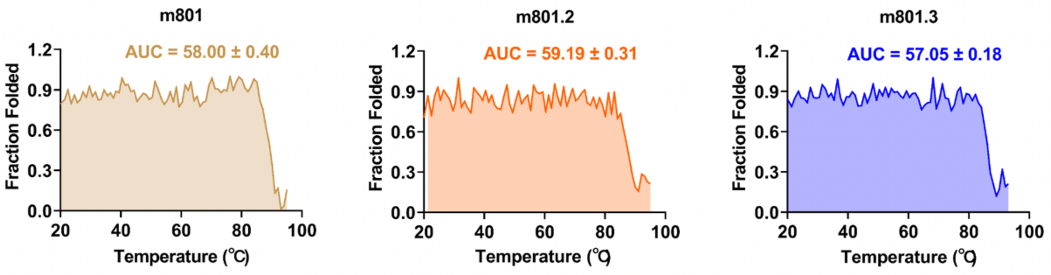

Figure S2 The area under the stability curve. Data are represented as mean ± SD.

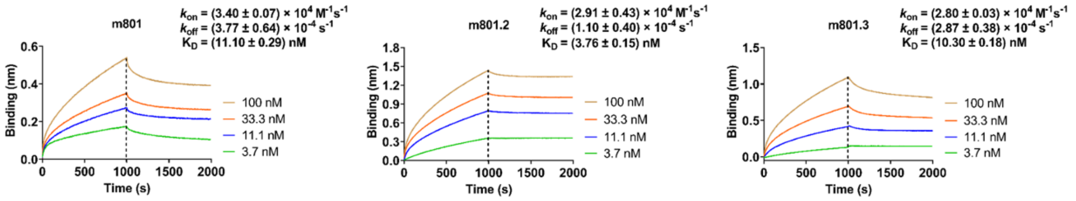

Figure S3 The binding affinities of m801, m801.2, and m801.3 to mouse EpCAM, as measured by BLI.

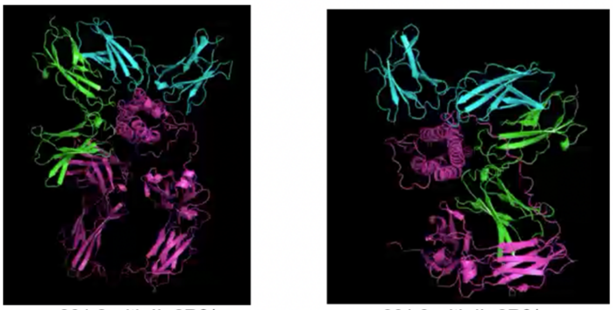

m801.2 with IL-2Rβ/γ   m801.3 with IL-2Rβ/γ

Figure S4 Molecular dynamics simulations for m801.2 and m801.3 with IL-2Rβ/γ complex. The green chain represents IL-2Rβ, the cyan chain represents IL-2Rγ, and the magenta chains represent either m801.2 or m801.3.

Original images:

Figure 2B

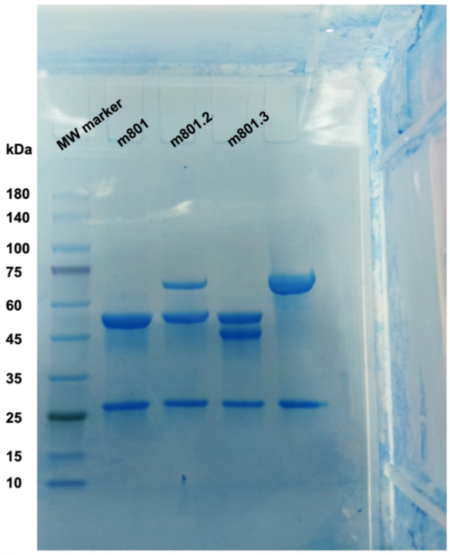

Supplementary Figure 1A

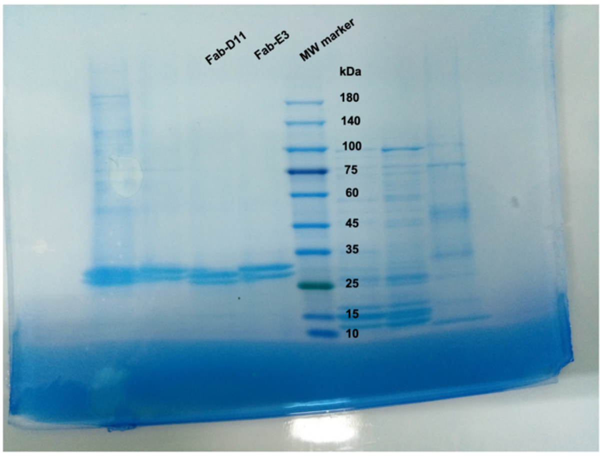

Supplement: Supplementary file 1 [file biomolecules-14-01399-s001.zip › biomolecules-3275527-supplementary.pdf]
